# Plant Uptake of Lactate-Bound Metals: A Sustainable Alternative to Metal Chlorides

**DOI:** 10.3390/biom11081085

**Published:** 2021-07-23

**Authors:** Lee J. Opdahl, Ricky W. Lewis, Lee A. Kalcsits, Tarah S. Sullivan, Karen A. Sanguinet

**Affiliations:** 1 Department of Crop and Soil Sciences, Washington State University, Pullman, WA 99164, USA; lee.opdahl@wsu.edu; 2 Department of Tropical Plant and Soil Sciences, University of Hawai’i at Mānoa, Honolulu, HI 96822, USA; ricky.w.lewis@gmail.com; 3 Tree Fruit Research and Extension Center, Washington State University, Wenatchee, WA 98801, USA; lee.kalcsits@wsu.edu

**Keywords:** metal lactates, hydroponics, micronutrients, biostimulants, organic acids, wheat, *A. thaliana*

## Abstract

Global agricultural intensification has prompted investigations into biostimulants to enhance plant nutrition and soil ecosystem processes. Metal lactates are an understudied class of organic micronutrient supplement that provide both a labile carbon source and mineral nutrition for plant and microbial growth. To gain a fundamental understanding of plant responses to metal lactates, we employed a series of sterile culture-vessel experiments to compare the uptake and toxicity of five metals (Zn, Mn, Cu, Ni, and Co) supplied in lactate and chloride salt form. Additionally, primary root growth in plate-grown *Arabidopsis thaliana* seedlings was used to determine optimal concentrations of each metal lactate. Our results suggest that uptake and utilization of metals in wheat (*Triticum aestivum* L.) when supplied in lactate form is comparable to that of metal chlorides. Metal lactates also have promotional growth effects on *A. thaliana* seedlings with optimal concentrations identified for Zn (0.5–1.0 µM), Mn (0.5–1.0 µM), Cu (0.5 µM), Ni (1.0 µM), and Co (0.5 µM) lactate. These findings present foundational evidence to support the use of metal lactates as potential crop biostimulants due to their ability to both supply nutrients and stimulate plant growth.

## 1. Introduction

Agricultural intensification to meet growing global food demand has prompted investigations into sustainable management practices, including alternative fertilization and application of biostimulants to enhance both crop growth and soil health, particularly in the face of global climate change [1,2,3,4]. In conventional agriculture, fertilization is largely focused on the three macronutrients nitrogen (N), phosphorus (P) and potassium (K), which has led to soil acidification and soils that are deficient in a range of micronutrients [5,6]. Micronutrients are essential for plant growth and development and often function as enzyme cofactors [7]. Micronutrient fertilizers are typically supplied in inorganic forms, such as chlorides or sulfate salts, to ameliorate deficiencies, but it is often unclear how such fertilizers impact additional processes within the soil ecosystem (e.g., microbial activity). While inorganic nutrients are rapidly available for plant uptake, they do not provide the additional benefits provided by organic fertilizers (e.g., green and livestock manure) through increased carbon (C) inputs. These valuable organic inputs from soil additions rich in a variety of C compounds have proven benefits such as increased overall soil health, soil water holding capacity, cation exchange capacity (CEC), aggregation and infiltration, pH stabilization, and enhanced microbial activities [8,9,10,11].

Organic amendments generally require microbial degradation to release nutrients into the soil solution for plant uptake, which is a relatively slow process, and the benefits are only realized long-term (a year, to several years past the current growing season). Thus, conventional chemical fertilizers are still required to obtain optimal yields [12,13]. A plausible alternative may be to supply micronutrients chelated to labile C compounds that can be rapidly metabolized by soil microbes and released into soil solution, thus stimulating microbial activity and providing readily available elements for plant nutrition. Furthermore, several studies have proven the ability of plant roots to directly take up small organic molecules such as low molecular weight organic acids (LMWOA), sugars, or amino acids, indicating that organic fertilizers of this nature could represent a direct source of nutrients for plant uptake [14,15,16,17,18].

When extracellular metal concentrations are high, they are preferentially accumulated in the roots or translocated to aerial tissues via xylem; the tendency to accumulate in the roots or shoots is primarily dependent on the specific metal and plant species [7]. For example, Cu is preferentially accumulated in the roots when external Cu concentrations are high, and thus plants under Cu toxicity usually exhibit reduced root growth [19]. However, high external Mn concentrations may manifest stress symptoms in aboveground tissues due to preferential accumulation of Mn in the shoots and leaves [7]. Thus, achieving the balance between micronutrient deficiency and toxicity is crucial to support optimal plant growth.

Metal lactates are an understudied class of organic micronutrient supplement that show potential to supply micronutrients and trace metals directly for plant uptake, in addition to providing a labile C source for soil and rhizosphere microbial populations. Previous work has shown lactic acid addition to soil enhances microbial activity [20,21], while foliar applications of lactic acid in hydroponically grown cucumber improves shoot development and yield [22,23]. In fact, lactic acid showed the greatest potential to enhance soil microbial activity in comparison to other LMWOA [20,24]. Lactic acid and lactate-conjugated metals are a common additive to vitamin supplements and widely used for food science applications [25]. However, little is known about the capacity of crops to uptake and translocate metals bound to lactate. In *Arabidopsis thaliana*, there are nine nodulin 26 intrinsic proteins (NIPs) that are highly conserved water and solute transport proteins. One of them, NIP2;1, was identified as a lactate transporter in roots induced particularly during periods of hypoxia and likely functioning in efflux [26]. Additionally, Zn/Fe permeases (ZIPs), heavy metal ATPases (HMAs), and Yellow Stripe Like (YSL) transporters, all have shown the ability to transport a range of metals [27,28,29].

The influence of lactate complexation on metal uptake and toxicity was assessed using five metals (Zn, Mn, Cu, Ni, and Co). The metals were supplied at toxic levels as either a lactate or a chloride salt form in aseptic hydroponic culture vessels, which allowed for comparison of the two forms of metals in the absence of microbial activity. Importantly, both metal chlorides and metal lactates have similar stability constants, making them suitable forms to compare on a chemical level [30,31,32]. The uptake capacity and subsequent toxicity of metal lactates was compared with that of metal chlorides to investigate the importance of plant uptake of the organic ligand-bound metals. Zn, Mn, Cu, Ni, and Co concentrations were measured in root and shoot tissues using MP-AES to validate uptake of each metal. To further investigate optimal metal lactate concentrations for plant growth, we generated dose−response curves for individual metal lactates by quantifying primary root growth rates in *A. thaliana* grown in a range of concentrations that more closely reflect physiologically relevant micronutrient levels according to previous reports [33].

## 2. Materials and Methods

### 2.1. Wheat Seed Sterilization and Pre-Germination

Wheat seedlings were cultivated under aseptic conditions, starting with seed sterilization prior to sewing in sterile culture vessels, and using sterile nutrient solutions. ”Louise” (*Triticum aestivum* L.) wheat seeds were chosen for this study due to the importance of this variety in the state of Washington [34]. Seeds were surface sterilized through a series of rinse steps adapted from Speakman and Kruger (1983) [35]. Briefly, 35 seeds were placed into 50 mL conical tubes and immersed in 40 mL of a 0.001% terramycin (CAS: 2058-46-0, Alfa Aesar, Haverhill, MA, USA) solution with 60 µL of 50% Tween 20 (23336-0010, Acros Organics, Fair Lawn, MA, USA) for 20 h at room temperature in the dark. Then, seeds were immersed in 40 mL of a 0.1% AgNO_3_ (CAS: 7761-88-8, Alfa Aesar, Haverhill, MA, USA) solution with 0.84 mL of 50% Tween 20 for 10 min. The AgNO_3_ solution was decanted and seeds were immersed in 40 mL of a 0.5% NaCl solution with 0.84 mL of 50% Tween 20 for 30 s. Seeds were rinsed five times with 40 mL of sterile Milli-Q (Milli-Q Advantage A10 System, Millipore Sigma, Burlington, MA, USA) water. After this sterilization procedure, seeds were placed on sterile Phytagel plates (0.4% Phytagel: P8169-250G, Sigma Life Science, St. Louis, MO, USA; 0.036% MgSO_4_: M63-500, Fischer Scientific; 0.018% CaCl_2_ dihydrate: C79-500, Alfa Aesar, Haverhill, MA, USA) and stored in the dark, at room temperature (~23 °C) for three days. After three days, pre-germinated seedlings were inspected for microbial contaminants, and only non-contaminated seedlings of approximately equivalent developmental stage were used in the study.

### 2.2. Wheat Cultivation Vessels and Media

To remove any possible residual and trace metals from experimental materials, all culture vessels, glass beads, and polypropylene pellets were acid-washed in a 100 mM HNO_3_ and HCl solution for a minimum of two hours and subsequently rinsed with ultrapure Milli-Q water eight times. Sterilized and pre-germinated wheat seedlings were grown for 13 days in 946 mL glass culture vessels (Phyto Technology Laboratories #C607, Lenexa, KS, USA) with vented screw caps (0.5 µm pore size; Phyto Technology Laboratories #C2055, Lenexa, KS, USA) to allow for gas exchange (Figure S1). Within each culture vessel, 250 g of 6 mm diameter glass beads were added and used as the physical plant growth support matrix. MilliQ water was then added according to volumes shown in Table S1, and each culture vessel was sterilized under 15 PSI at 121 °C for 30 min. A 0.25× modified Hoagland’s nutrient solution (pH 5.9) described in Table S2 was added to each culture vessel in the volumes indicated in Table S1 [36]. Metals and nutrients that were added as a part of the experimental design in the form of either lactate or chloride treatments that would normally be included in a complete Hoagland’s nutrient solution (i.e., Zn, Cu, and Mn), were excluded from the Hoagland’s solution for that treatment. A 0.229 M stock solution of each metal lactate (Zn, Cu, Mn, Ni, and Co lactate) and metal chloride (Zn, Cu, Mn, Ni, and Co chloride) was prepared. Metal chloride solutions were sterilized by autoclaving at 15 PSI, 121 °C for 30 min, while the metal lactate solutions were filter sterilized (Millipore Sigma #SCVPU11RE, Burlington, MA, USA; 0.1 µm pore size). Metal lactate and metal chloride solutions were each added in the volumes shown in Table S1 to bring total solution volumes in each vessel to 50 mL to create the final treatment concentrations also shown in Table S1. The treatment concentrations used in this study were chosen to reflect concentrations that are several orders of magnitude higher than the original concentration of Mn, Cu, and Zn in Hoagland’s nutrient solution. Since Co and Ni are not included in a complete Hoagland’s nutrient solution, the same concentrations were used across all treatments. Control culture vessels did not receive the metal added via metal lactate and metal chloride treatments.

Following the nutrient solution preparation, each sterile wheat seedling was carefully placed into the glass beads, and the surface of the glass beads in each vessel was then covered by a layer of sterile, black polypropylene pellets to prevent excessive light exposure on seedling roots. Additionally, black plastic covers (ShuBee, Macon, GA, USA) were placed on the bottom exterior portion of each culture vessel to further inhibit light exposure on roots. Wheat seedlings were grown in culture vessels at room temperature under growth lights (Ecolux w/Starcoat, F32T8 SPX35 Eco, GE, Boston, MA, USA; photon flux of 55 µmol m^−2^ s^−1^) with a photoperiod of 16 h for 13 days.

### 2.3. Microbial Contamination Analysis

Prior to collecting each wheat seedling for growth and tissue analysis, a 100 mL aliquot of nutrient solution from each culture vessel was inoculated onto sterile 0.8% tryptone yeast agar (tryptone: 0123-17, Difco Laboratories, Detroit, MI, USA; yeast extract: J850-500G, Amresco Inc., Solon, OH, USA; agar: J637-500G, VWR International, Radnor, PA, USA) plates to detect culturable microorganisms. The plates were stored in the dark at room temperature for a minimum of six days and visually inspected for any microbial colonies. Plates displaying microbial growth represented culture vessels that were contaminated, and those vessels were not included in the plant growth parameter results.

### 2.4. Wheat Growth and Metal Response

At the conclusion of the 13-day growth period, each wheat seedling was carefully removed from the culture vessel, and the roots were washed in a 0.01 M CaCl_2_ solution to remove loosely bound metals from the root surface. The seedlings were then cut above the seed crown to separate the shoot and root tissues, and shoot tissues were measured for total length (cm), and both the shoot and root tissues were weighed to obtain fresh weights (FW). Tissues were then stored in 50 mL conical tubes, flash-frozen in liquid nitrogen, and subsequently stored at −80 °C. Frozen tissues from the control (0 mM), 0.343, and 3.43 mM treatments were lyophilized using a Labconco FreeZone 6 freeze dryer (Labconco Corporation, Kansas City, MO, USA) for at least 72 h and subsequently weighed to obtain dry weights (DW).

### 2.5. Wheat Tissue Analysis

Below and above ground lyophilized tissue were weighed into PTFE digestion tubes. Because these were small samples, the entire sample (1–30 mg) was added to the tubes and acid digested using 1 mL of HNO_3_. After digestion, 1 mL of ultrapure water was added to each vial. The solutions were filtered with a 0.45 µm PTFE filter (Thermo Fisher Scientific, Waltham, MA, USA). Filtered digests were then diluted 25× and analyzed using an Agilent 4200 microwave plasma-atomic emission spectrometer (MP-AES) (Agilent, Santa Clara, CA, USA) and run in combination with Zn, Cu, Mn, Co, and Ni ICP standards ranging from 0–500 ng g^−1^ for validation.

### 2.6. Plate-Growth Assays

A series of plate-growth assays were used to determine optimal concentrations for each individual metal lactate. *Arabidopsis thaliana* (ecotype Columbia-0) seeds were surface sterilized in a 10% (*v*/*v*) bleach and 0.02% (*v*/*v*) Triton X-100 solution for 5 min followed by a 1-min wash in 70% (*v*/*v*) ethanol. Seeds were rinsed with sterile nanopure water before and after the ethanol wash. Sterilized seeds were transferred onto 1% Bacto agar plates (pH 5.8) containing 0, 0.5, 1, 50, and 75 µM of individual metal lactate and 0.25× modified Hoagland’s nutrient solution. Metals that were added as metal lactates that would normally be included in a complete Hoagland’s nutrient solution (i.e., Zn, Cu, and Mn), were excluded from the Hoagland’s solution for that treatment. Each concentration was represented by 8–10 sterilized seeds per plate on four plates. Plates were sealed with micropore tape and placed in a dark 4 °C refrigerator for 2–4 days for cold stratification. The trial was repeated in triplicate for each metal lactate.

Following cold stratification, plates were transferred to a Percival Model CU22L growth chamber (~100 µmol m^−2^ s^−1^) where they were maintained at 24 °C. After the first 24 h of incubation, an initial mark was made on the exterior of the plate just over the top of the tip of the primary root (PR). Plate marking was repeated every 24 h for a total of 5 days after the initial mark to monitor daily PR growth. Plates were scanned using an Epson V700 flatbed scanner, and the scanned images were imported into ImageJ software (Java 8.0) to quantify daily PR growth.

### 2.7. Statistical Analyses

In the wheat cultivation vessel experiment, a *t*-test was used to identify differences between metal chloride and metal lactate treatments within each concentration for each plant growth parameter (Excel Version 2104, Microsoft, Redmond, WA, USA). A Fischer’s Least Significant Difference test was used to discern differences in nutrient content between concentrations within the same metal delivery treatment using the R statistical computing environment (R Development Core Team, 2017). Correlations between treatment and tissue metal concentrations were analyzed using a Pearson correlation test in the R statistical computing environment. In the plate-based assay experiment, differences in daily PR growth between metal lactate concentrations were assessed using a Tukey’s Honest Significant Difference test in the R statistical computing environment.

## 3. Results

### 3.1. Wheat Cultivation Vessel Experiment and Arabidopsis thaliana Plate-Growth Assays

To test the hypothesis that wheat seedlings can utilize metal lactates as effectively as metal chlorides, and therefore experience metal toxicity at the same rate, a series of sterile culture-vessel experiments were employed. Wheat seedlings were exposed to varying levels of each of the metal lactates and metal chlorides, and growth parameters as well as tissue metal concentrations were analyzed. Plant growth and tissue metal content were analyzed to evaluate the degree of toxicity as an indicator of total metal uptake in wheat. In accordance with our hypothesis, plant growth generally decreased as the metal concentrations increased regardless of lactate or chloride metal (Tables 1, 3, 5, 7 and 9). Increasing metal concentrations led to an increase in tissue metal content (Tables 2, 4, 6, 8 and 10) at the 0.343 mM and 3.43 mM concentrations. Tissue metal content was not calculated for the 34.3 mM and 100 mM concentrations due to the level of toxicity and insufficient amounts of tissue required for reliable MP-AES analysis. Moreover, although the mean metal content increases at higher metal concentrations, this increase was in some cases non-significant due to the high variance of uptake between individual plants at these toxic concentrations. Furthermore, primary root growth measurements of *Arabidopsis thaliana* seedlings grown on plates varying in metal lactate concentrations allowed us to identify optimal concentration ranges for each individual metal lactate. This is a necessary step to inform future work in characterizing metal lactates since no data on the subject has been published to date.

#### 3.1.1. Zinc

Typically, concentrations of Zn in plant tissues range from 10–120 µg g^−1^ dw, whereas critical toxicity concentrations range from 100–300 µg g^−1^ dw depending on plant species, age, and other nutrient concentrations [7,37,38]. Overall, we found that all plant growth parameters generally decreased with increasing Zn concentrations, regardless of the form of metal ligand, lactate or chloride (Figure S2). Within each treatment level, no significant differences between ZnCl_2_ and Zn lactate were observed in any of the plant growth parameters, with the exception of shoot length at 34.3 mM. Mean shoot growth was 4.5 ± 1.4 cm and 2.8 ± 0.7 cm for Zn lactate and ZnCl_2_, respectively (Table 1). Uptake of Zn from both delivery methods is supported by the linear increase of Zn in root and shoot tissues corresponding with the increased Zn in the nutrient solution (Table 2). Two significant negative trends were observed between Mn and Zn in which Mn tissue concentrations decreased in the roots with increasing ZnCl_2_ supply (*R* = −0.53, *p* = 0.042), and in the shoots with increased Mn lactate (*R* = −0.61, *p* = 0.015). ZnCl_2_ supply resulted in significantly higher root tissue accumulation of Zn at 11,975.6 ng g^−1^ while Zn lactate was lower at 6729.1 ng g^−1^, when both were applied at 3.43 mM.

**Figure 1:**
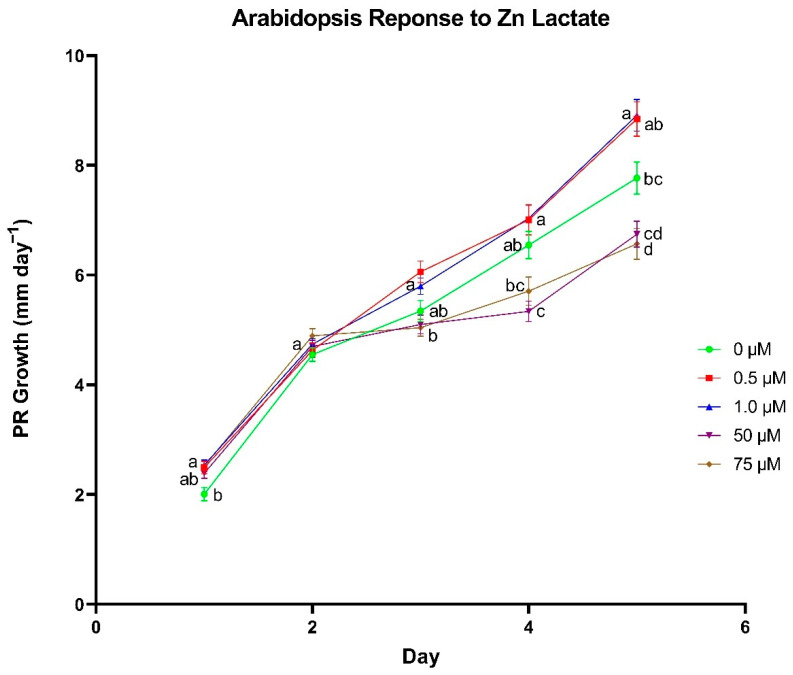
The effect of zinc (Zn) lactate concentration on primary root (PR) growth rate in *Arabidopsis thaliana*. Differences between means were compared using Tukey’s Honest Significant Difference test, where different letters indicate a statistically significant difference between treatment concentrations in their respective day of growth. Error bars denote means ± SE (*n* = 53–78).

To further assess the potential growth promoting effects of Zn lactate, we used primary root growth in *A. thaliana* as an indicator of plant responsiveness to Zn lactate. A range of 0.5–1 µm Zn lactate was found to promote root growth (Figure 1). The response to Zn lactate supplementation was observed in the first day of growth, where 0.5 and 1.0 µm led to an average PR growth rate of 2.49 and 2.53 mm day^−1^, respectively, whereas seedlings that did not receive Zn lactate averaged 2.0 mm day^−1^. Beginning on day 3 of growth, seedlings treated with 50 and 75 µm had significantly reduced growth rates compared to those treated with 0.5 and 1.0 µm. For example, we see the greatest difference on the fifth day of growth where seedlings treated with 50 µm averaged 6.74 mm day^−1^ and those treated with 75 µm were slightly lower at 6.57 mm day^−1^, while 0.5 and 1.0 µm treatments averaged 8.84 and 8.91 mm day^−1^, respectively. Furthermore, both 0.5 and 1.0 µm treatments on day 5 conferred significantly greater growth compared to seedlings that did not receive Zn lactate with an average root growth rate of 7.77 mm day^−1^.

#### 3.1.2. Manganese

Adequate Mn concentrations in plant tissues typically range from 90–200 µg g^−1^ dw, whereas critical toxicity concentrations of Mn range from 200–5300 µg g^−1^ dw depending on a variety of plant factors [7,38]. Our results show that growth decreased as MnCl_2_ and Mn lactate supply increased. Only one significant difference was identified in shoot FW at the 3.43 mM treatment level where shoot FW was 209.8 ± 21.7 mg when Mn lactate was added compared to an average of 147.6 ± 46.1 mg from MnCl_2_ supplementation (Table 3). Tissue metal analysis confirmed an increase in Mn uptake with increasing Mn supply from both ligand forms (Table 4). No significant differences in tissue metal concentrations were detected between MnCl_2_ and Mn lactate at any concentration for any metal analyzed.

**Figure 2:**
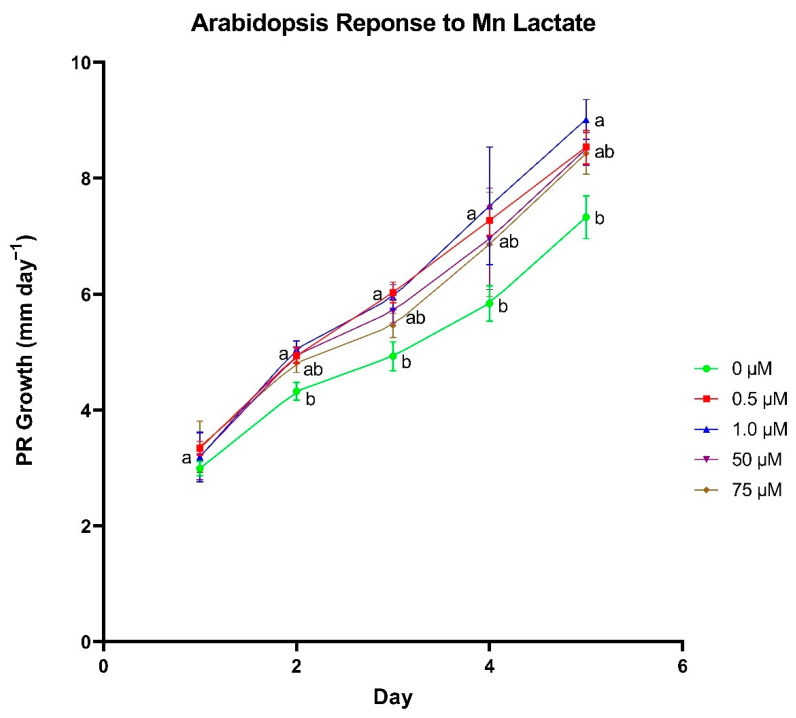
The effect of manganese (Mn) lactate concentration on primary root (PR) growth rate in *Arabidopsis thaliana*. Differences between means were compared using Tukey’s Honest Significant Difference test, where different letters indicate a statistically significant difference between treatment concentrations in their respective day of growth. Error bars denote means ± SE (*n* = 51–63).

Primary root (PR) growth rates of *A. thaliana* seedlings were highest when 0.5 and 1.0 µm Mn lactate was added (Figure 2). While no differences in growth rates were observed after the first day, we observed differential growth rates in seedlings on day 2. Seedlings treated with 0.5, 1.0 and 50 µm Mn lactate averaged 4.93, 5.05 and 4.95 mm day^−1^, respectively. PR growth rates were the lowest at 4.32 mm day^−1^ when seedlings did not receive Mn lactate. The difference in PR growth rates between seedlings not supplemented with Mn lactate and those treated with 0.5 and 1 µm Mn lactate increased in days 3−5. For example, on day 4, seedlings treated with 0.5 and 1.0 µm averaged 7.27 and 7.52 mm day^−1^, whereas PR growth rates averaged just 5.84 mm day^−1^ in seedlings with no Mn lactate supplementation. PR growth rates on day 5 were similar to day 4; however, there was no statistical difference between the 0.5 µm treatment and the 0 µm control. Although no difference was identified, seedlings supplemented with 0.5 µm Mn lactate grew approximately 1.5 mm more on average compared to the control.

#### 3.1.3. Copper

Compared to Zn and Mn, normal tissue concentrations of Cu are generally lower at around 10–25 µg g^−1^ dw depending on factors such as plant species, age, and other nutrient levels [7,38]. In light of this, we saw greater plant growth inhibition with increasing treatment concentrations of both Cu delivery forms compared to Zn and Mn treatments. No differences in growth parameter measurements were observed between CuCl_2_ and Cu lactate treatments except for root DW at the 3.43 mM treatment. At that concentration, root DW was lower when CuCl_2_ was applied (2.0 ± 1.0 mg) compared to Cu lactate (3.3 ± 0.4 mg) (Table 5). Increasing solution concentrations of both Cu forms led to an increase in tissue Cu levels in both roots and shoots. No significant differences in tissue metal concentrations were detected between CuCl_2_ and Cu lactate at any solution concentration for any metal analyzed (Table 6).

**Figure 3:**
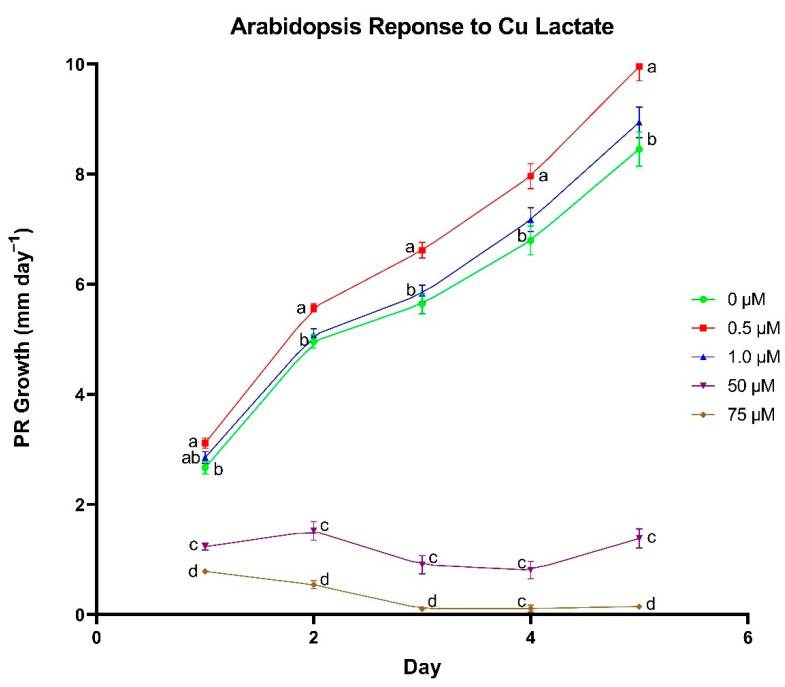
The effect of copper (Cu) lactate concentration on primary root (PR) growth rate in *Arabidopsis thaliana*. Differences between means were compared using Tukey’s Honest Significant Difference test, where different letters indicate a statistically significant difference between treatment concentrations in their respective day of growth. Error bars denote means ± SE (*n* = 60–82).

At a concentration of 0.5 µm, Cu lactate significantly enhanced primary root growth rate in *A. thaliana* in each of the first 5 days of growth compared to seedlings grown in 0 µm conditions (Figure 3). In addition, the difference in growth rate between seedlings treated with 0.5 µm and those treated with 0 µm increased as the length of growing time increased, where the growth rate difference for days 1–5 was 0.45, 0.62, 0.97, 1.17 and 1.5 mm day^−1^, respectively. Supplementing Cu lactate at a concentration of 1.0 µm had no difference in PR growth rates compared to the control, and 50 and 75 µm treatments yielded a negative effect on PR growth.

#### 3.1.4. Nickel

The adequate range for Ni in plant tissue is typically between 0.05–10 µg g^−1^ dw with critical levels being as high as 100 µg g^−1^ dw [7]. Plant growth parameter results revealed five differences between Ni ligand forms. At the 0.343 mM treatment level, Ni lactate supplementation averaged 27.1 cm of shoot growth and 221.7 mg of shoot FW, but averaged 19.5 cm and 139.4 mg, respectively, with NiCl_2_ (Table 7). Similarly, we observed differences in shoot length and shoot FW between Ni forms at a solution concentration of 100 mM, where shoot length (3.3 ± 0.4 cm) and FW (22.9 ± 1.7 mg) were higher when Ni lactate was used compared to NiCl_2_ (2.3 ± 0.4 cm and 12.6 ± 2.1 mg, respectively). Shoot growth was higher at the 34.3 mM treatment level when Ni lactate was applied (3.4 ± 0.8 cm) compared to NiCl_2_ (2.1 ± 0.6 cm). Differences in Ni tissue concentrations between Ni delivery forms were observed in roots at a solution concentration of 3.43 mM and in shoots at 0.343 mM. In the root at 3.43 mM, Ni content from Ni lactate was approximately five times less than Ni supplied via NiCl_2_; however, no significant differences were observed in plant growth metrics at this treatment level (Table 8). Seedlings supplemented with 0.343 mM Ni lactate had lower Ni content (252.0 ± 28.4 ng g^−1^) in the shoots compared to NiCl_2_ (598.6 ± 32.9 ng g^−1^).

**Figure 4:**
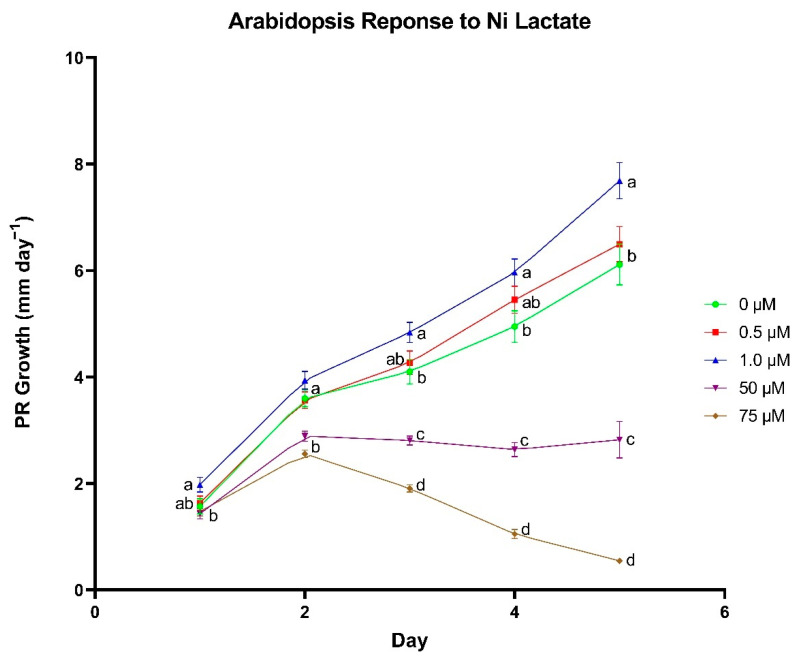
The effect of nickel (Ni) lactate concentration on primary root (PR) growth rate in *Arabidopsis thaliana*. Differences between means were compared using Tukey’s Honest Significant Difference test, where different letters indicate a statistically significant difference between treatment concentrations in their respective day of growth. Error bars denote means ± SE (*n* = 34–95).

PR growth rates for *A. thaliana* were the highest when nickel lactate was supplied at 1.0 µm (Figure 4). In the first two days no difference in PR growth was observed between control seedlings and seedlings treated with 1.0 µm Ni lactate. Starting on day 3, root growth rates slowed when nickel was applied at higher concentrations (50 and 75 µm). Seedlings treated with 1.0 µm Ni lactate had a significantly higher growth rate at 4.83 mm day^−1^ compared to mean growth rates for control seedlings (4.10 mm day^−1^). The differences between treatments continued to increase on days 4 and 5. No difference was observed in daily PR growth rate between control seedlings and seedlings grown in 0.5 µm Ni lactate, and 50 and 75 µm treated seedlings had a negative effect on PR growth.

#### 3.1.5. Cobalt

The majority of studies investigating Co concentrations in plant tissues have been done on Co hyperaccumulating plants to better understand tolerance mechanisms in metalliferous soils [39]; however, the critical toxicity concentration of Co in crop and pasture species (e.g., clover, bean and cabbage) has shown to range from 0.4 µg g^−1^ dw to a few µg g^−1^ dw depending on the plant species [7,40,41]. Significant differences between the Co lactate and CoCl_2_ treatments were identified in the shoot length and root DW parameters (Table 9). Shoot length was greater when Co lactate was applied at 34.3 mM and 100 mM (2.6 ± 0.4 cm and 1.4 ± 0.2 cm, respectively) compared to when CoCl_2_ was applied (2.1 ± 0.3 cm and 1.0 ± 0.3 cm, respectively). Root DW was greater when Co lactate was applied at 3.43 mM concentrations (6.4 ± 1.4 mg) compared to 4.3 ± 1.1 mg for CoCl_2_ at the same concentration. While no significant differences in tissue metal concentrations were detected between Co ligand forms at any concentration for any metal analyzed, there is a general decrease in the uptake and concentration of Cu, Mn, Ni, and Zn in the root and shoot tissues as Co solution concentration increases from both Co forms (Table 10).

**Figure 5:**
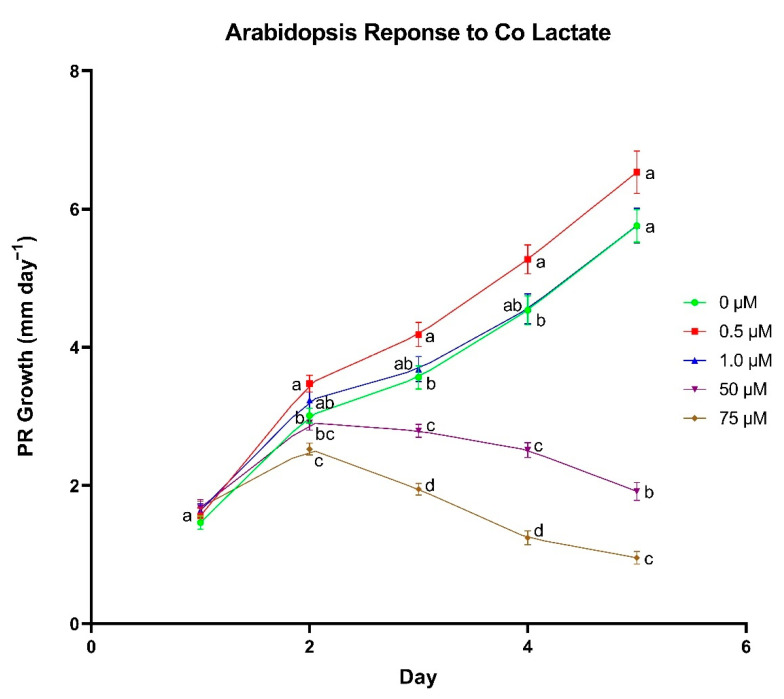
The effect of cobalt (Co) lactate concentration on primary root (PR) growth rate in *Arabidopsis thaliana*. Differences between means were compared using Tukey’s Honest Significant Difference test, where different letters indicate a statistically significant difference between treatment concentrations in their respective day of growth. Error bars show means ± SE (*n* = 40–74).

*A. thaliana* primary root growth rate was enhanced by the addition of 0.5 µm Co lactate (Figure 5). On day 2, 0.5 µm treated seedlings had a significantly higher growth rate at 3.47 mm day^−1^ compared to 3.01 mm day^−1^ in control seedlings for a difference of 0.46 mm day^−1^. The difference in growth rate between control seedlings and 0.5 µm treated seedlings increased slightly on days 3–5 of growth to 0.62, 0.74 and 0.78 mm day^−1^; however, the difference between these two groups was not significant on day 5. There was no difference in primary root growth rate between the control and seedlings supplemented with 1.0 µm Co lactate, whereas 50 and 75 µm treatments appeared to have a significant inhibitory effect on root growth.

## 4. Discussion

This study provides the first investigation characterizing metal lactates as a potential plant biostimulant. To the authors’ knowledge, metal lactates have only previously been studied as a feed additive for ruminants to determine their effects on nutrient digestibility, in which Co lactate has been shown to enhance bacterial fiber digestion compared to CoCO_3_ and promote microbial volatile fatty acid production in cattle [42,43]. The proven enhancement of microbial activity driven by metal lactates in the digestive tract of ruminants suggests a similar phenomenon might be observed in the soil ecosystem; however, there is no information on plant uptake or optimal concentration of lactate-bound metals. This information is not only essential for further studies on metal lactates, but it also provides baseline evidence that metal lactates have biostimulatory effects on plants. Furthermore, when characterizing novel crop biostimulants it is apropos to first define the plant response, especially for micronutrient-containing biostimulants, since the range between deficiency and toxicity in plant micronutrient levels tends to be narrow [7]. The current study was conducted to address the dearth of information regarding metal lactate−plant interactions.

The first objective was to compare the uptake and toxicity range of five metals (Zn, Mn, Cu, Ni, and Co) supplied as either a lactate or a chloride salt form, to determine uptake in the absence of microbial breakdown processes using wheat grown in sterile culture vessels. Based on plant growth metrics and tissue metal content, metals supplied in lactate form appear to have a similar affinity for plant uptake compared to metals in chloride form. A previous study by Neocleous et al. (2020) found similar results when comparing the impact of chelated and inorganic forms of Mn and Zn on plant performance metrics and nutrient uptake in hydroponically grown bean crops [44]. In their study, bean plants receiving Mn and Zn chelated with ethylenediaminetetra acetic acid (EDTA) showed negligible differences in tissue micronutrient content compared to those receiving sulfate mineral forms. Chelating agents such as EDTA are often used to enhance plant micronutrient uptake in both soil and soilless growing systems by protecting them from redox changes and/or precipitation, thereby making them more available in solution [45]. Similarly, LMWOA (e.g., acetic, malic, and oxalic acid) also serve as small, organic complexing molecules and have been shown to increase heavy metal uptake in hydroponically grown wheat [46,47,48]. Although the precise mechanism by which complexed metals are taken up by plant roots is still under investigation, the data indicate they do play a beneficial role in facilitating metal uptake in plants. Based on the work from Neocleous et al. (2020), as well as the findings from the present study, it appears that organometallic complexes may be as effective in delivering metal nutrients compared to mineral forms, at least in hydroponic systems [44]. Further investigation is required to determine the efficacy of metal lactates to supply plants with metal nutrients in more complex soil systems.

A negative trend was observed between Mn and Zn where Mn tissue concentrations decreased with increasing ZnCl_2_ and Zn lactate concentrations. Lewis et al. (2012) observed Zn treatment increased Mn translocation in nodulated *Medicago truncatula* plants [49], and others have observed that increased Zn treatment is associated with decreased Mn tissue concentrations in various plants including sunflower, wheat, and lemon balm (*Melissa officinalis*) [50,51,52]. In peanut (*Arachis hypogaea* L.), Zn and Mn uptake are facilitated by the transporter AhNRAMP1, which could indicate that excessive Zn supply may suppress Mn uptake as a result of competition for transporters in root cells, thus providing a potential explanation for the findings presented above [53]. Interestingly, our findings suggest there is a difference in translocation of Zn depending on the form in which it is supplied. This could indicate that Zn supplied in lactate form may be regulated by different transporters than Zn ions from chloride forms once inside the plant; however, further investigation is needed to elucidate potential differences and mechanisms involved in the translocation and partitioning of metals in plants when supplied these two forms.

To further investigate the physiological responses to metal lactates beyond uptake capacity and toxicity thresholds, we identified concentrations for each individual metal lactate that yielded optimal root growth in plate-grown *A. thaliana* seedlings. This allowed us to gauge nutrient status and concentration as a result of plant uptake as has been described previously [33]. Root growth measurements have frequently been used as an indicator for responsiveness to fertilizer treatments in a variety of plant species [54,55]. While root growth can be quantified in different ways (e.g., growth rates, density, and biomass), root growth promotion in any aspect generally correlates to increased plant vigor through numerous mechanisms including enhanced nutrient and water acquisition and heightened tolerance to abiotic and biotic stressors [56,57,58]. Our finding that metal lactates delivered at specific concentrations can enhance primary root growth in *A. thaliana* seedlings shows promise in their ability to promote overall plant health.

The ability of metal lactates to improve primary root growth in *A. thaliana* seedlings may be attributed, at least in part, to enhanced activity in the enzymes and cellular processes that require micronutrients. Likewise, microbial growth and activity may also be stimulated by metal lactates by providing metal nutrients required for metabolic processes. For example, Zn in plants and microbes is primarily involved in the functionality and structural integrity of enzymes and proteins. Some of the more well-characterized plant enzymes and proteins requiring Zn include carbonic anhydrase, the Zn-containing superoxide dismutase (SOD), and ribosomal proteins, which have roles in C fixation, detoxification of reactive oxygen species, and protein synthesis, respectively [59,60,61,62,63]. Zinc supplementation has been shown to enhance the activity of carbonic anhydrase [61] and the Zn-containing SOD [64], as well as increase protein synthesis by mitigating ribosomal disintegration [65], and in each case plants receiving Zn had greater overall growth compared to Zn-deficient plants. Microbial alkaline phosphatase is particularly important in crop production settings due to its catalytic function in cleaving assimilable phosphate from organic P sources [66]. Zinc is a cofactor required for microbial alkaline phosphatase activity; however, a report by Łukowski and Dec (2018) found acid-soluble and exchangeable fractions of Zn to be negatively correlated with alkaline phosphatase activity [67]. Interestingly, the same study found a significant positive correlation between Zn and dehydrogenase activity, which is generally used as an indicator of overall biological activity [68].

Manganese mainly serves as a cofactor, activating about 35 different enzymes primarily involved in oxidation-reduction, decarboxylation, and hydrolytic reactions [7,69]. For example, Mn is a cofactor for phenylalanine ammonia-lyase and peroxidase which are enzymes involved in lignin biosynthesis [70]. Manganese deficiency is shown to reduce lignin content in plant roots [71], which has been linked to decreased resistance against root-infecting pathogens due to the role of lignin in pathogen defense [72]. Microbial Mn peroxidase also has an absolute requirement for Mn and is an ecologically important enzyme involved in the degradation of lignin, and thereby contributes to the recycling of more recalcitrant plant-derived C in soils [73,74]. The activity of Mn peroxidase appears to be enhanced by high concentrations of Mn which subsequently leads to greater lignin breakdown [75].

Currently, more than 100 different Cu-containing proteins have been identified in plants including polyphenol oxidase and diamine oxidase [76]. Polyphenol oxidases catalyze the oxygenation reactions converting plant phenols to orthoquinones, which are important precursors in the biosynthesis of lignin and in the production of melanotic substances that are formed from tissue damage in crops such as in potatoes and apples [77]. In addition to the role of lignin in pathogen defense, melanotic compounds act as inhibitors of spore germination and fungal growth [7]. Diamine oxidase is involved in the degradation of the nitrogen-containing compounds putrescine and spermidine, producing hydrogen peroxide and ammonia as byproducts of catalysis [78]. Hydrogen peroxide produced during these reactions is thought to be involved in cell signaling, pathogen defense, and increasing the activity of peroxidases associated with lignification [79,80]. The activity of both polyphenol oxidase and diamine oxidase have shown clear responses to Cu supplementation, wherein Cu-sufficient plants had higher activities of the enzymes and greater plant biomass compared to plants grown in Cu-deficient conditions [81]. In microbes, Cu is required by several enzymes including laccase and polysaccharide oxygenase which function in the breakdown of lignin and cellulose, respectively [82,83]. The activity of both enzymes can be enhanced by Cu supplementation in culture-based setups [82,84]; however, further investigation is needed to determine how Cu amendments may improve lignin and cellulose degradation in soils.

To date, only two plant proteins are known to require Ni for proper functioning: urease and Ni-urease accessory protein (Eu3). Urease catalyzes the hydrolytic reaction converting urea into ammonia and carbon dioxide, and Ni-urease accessory protein is essential for urease activity [85,86]. In studies using urea as the major or sole source of nitrogen, Ni-sufficient plants showed a significant increase in growth and urease activity compared to Ni-deficient plants [87,88,89]. Likewise, Ni is required for urease function in microbes and the activity of the enzyme can be inhibited in Ni-deficient soils as well as stimulated upon Ni supplementation [90].

Cobalt, although not required for plant growth, is considered a beneficial element that can enhance growth, particularly in leguminous plants, due to its requirement by nitrogen-fixing symbionts [39]. For example, in *Rhizobium* and *Bradyrhizobium*, Co-dependent cobalamin is required for the synthesis of methyl malonyl-coA mutase which is involved in leghemoglobin synthesis in root nodules, wherein leghemoglobin is crucial to the protection of nitrogenase from oxygen [91]. The absence of Co in nodule-forming plants can lead to reduced bacteroid numbers in root nodules and reduced nodule formation, ultimately decreasing nitrogen fixation [7,92]. This is particularly important in plants relying on microbial nitrogen fixation as a source of N when mineral or other forms are in low abundance or unavailable.

The importance of micronutrients in crop production is gaining awareness as micronutrient-deficient soils are becoming increasingly prominent across the globe [5]. Generally, conventionally managed soils tend to have lower micronutrient availability compared to soils under organic management which can disrupt or inhibit plant metabolic processes. Organic cropping systems tend to procure sufficient levels of soil micronutrients which is likely due, in part, to the use of organic fertilizers and amendments that supply C to soil microbial communities. Organic C availability in soils is directly linked to soil microbial activity, wherein soils with high amounts of available C generally favor microbial growth and plant growth promoting functions such as P solubilization, siderophore production, phytohormone production, and biocontrol activities [93,94]. A variety of C amendments have been applied to soils with the goal of enhancing microbial functions including C supplied in the form of rhizospheric organic acids. In their report, Macias-Benitez et al. (2020) determined that organic acids, particularly lactic and citric acid, are effective in stimulating soil microbial activity and enhancing bacterial taxa described as plant growth promoting bacteria [24]. Along with our work, the findings by Macias-Benitez et al. (2020) provide substantial evidence supporting the use of metal lactates as potential biostimulants.

In addition to serving as a C source of microbial nutrition, lactate is a key primary metabolite and may serve a beneficial role in early seedling growth and development. In our study, we identified several differences in plant growth between metal lactate and metal chloride treatments at similar concentrations, and in each case, metal lactates had less of an inhibitory effect (Tables 1, 3, 5, 7 and 9). For example, Ni lactate supplied at a rate of 0.343 mM led to significantly higher shoot FW compared to NiCl_2_ treatments at the same concentration (Table 7). Carbon sources are often used in in vitro plant cultures and serve as an energy and C source for highly demanding developmental processes such as embryogenesis, organogenesis, shoot proliferation, and root induction since developing seedlings grown in culture are not entirely autotrophic [95]. The most widely used C source for growing in vitro plants is the disaccharide sucrose; however, a variety of other carbohydrates including other disaccharides, monosaccharides, trisaccharides, and sugar alcohols are also applied depending on the plant species and growth stage. Organic acids are typically not used for this purpose, but rather they have been incorporated in in vitro culture to facilitate metal nutrient uptake due to their role as a complexing agent. For example, lactic acid salts in hydroponic solution have been shown to significantly improve cucumber development and yield in nutrient-deficient conditions compared to non-deficient conditions without lactic acid salts [22]. In addition to its role in enhancing metallonutrient uptake, the finding that Co lactate enhanced *A. thaliana* root growth in our study suggests that lactate may fulfill the same role as carbohydrates to supply *A. thaliana* seedlings with the C and energy, considering they were grown in nutrient sufficient conditions and the unlikelihood that Co supplementation benefitted growth and development.

## 5. Conclusions

We present here an initial characterization of metal lactates as a potential plant biostimulant. Our data shows a comparable ability for wheat seedlings to utilize metals (i.e., Zn, Mn, Cu, Ni, and Co) delivered in lactate form compared to mineral chloride forms. Moreover, metal lactates promote root growth on plate-grown *A. thaliana* seedlings in the first five days of growth with optimal concentrations for Zn (0.5–1.0 µM), Mn (0.5–1.0 µM), Cu (0.5 µM), Ni (1.0 µM), and Co (0.5 µM) lactate being identified. In a push for sustainable fertilization alternatives in the face global climate change, metal lactates show promise as a novel class of biostimulants that may provide additional benefits to cropping systems that are not offered by conventional fertilizers. Previous work showed metal lactates can promote microbial activities in animals. Given that crop micronutrient deficiency often occurs in areas where soils have been heavily degraded, metal lactates might provide a unique solution by providing adequate micronutrient fertilization while also promoting soil health. To further characterize metal lactates as a potential plant biostimulant, future studies will work toward describing the distribution of metals provided by metal lactates in soil systems, the effect of metal lactates on gene expression in plants, as well as their effects on soil microbial communities and activity. The findings presented here lay the foundation for such studies as well as subsequent investigations on the ability of metal lactates to enhance plant growth and nutrition.

## Figures and Tables

**Table 1:** Plant growth parameter results for zinc (Zn) treatments. After 13 days of growth, wheat seedlings were harvested, and shoot length was measured along with fresh weight (FW) and dry weight (DW) of both root and shoot tissue. A *t*-test was used to identify differences between zinc chloride and zinc lactate treatments within each treatment level for each plant growth parameter, where superscripts “a” and “b” denote treatment differences of *p* < 0.05. ±: standard deviation.

Treatment Conc.	Treatment	Shoot Length (cm)	Shoot FW (mg)	Shoot DW (mg)	Root FW (mg)	Root DW (mg)
0 mM	Control	35.7 ± 19.4	358.6 ± 48.0	36.6 ± 5.9	186.7 ± 45.6	11.4 ± 2.4
0.343 mM	ZnCl_2_	35.2 ± 0.7	303.0 ± 53.6	33.2 ± 3.9	155.6 ± 33.2	13.5 ± 3.8
	Zn Lactate	34.7 ± 1.5	299.3 ± 54.1	32.1 ± 7.6	140.3 ± 52.5	10.2 ± 5.4
3.43 mM	ZnCl_2_	14.6 ± 7.4	104.6 ± 52.9	17.9 ± 9.1	47.1 ± 14.1	6.9 ± 0.7
	Zn Lactate	18.5 ± 2.8	149.6 ± 19.3	26.9 ± 6.5	42.1 ± 10.2	9.4 ± 5.6
34.3 mM	ZnCl_2_	2.8 ± 0.7 ^a^	17.4 ± 4.8		26.0 ± 4.7	
	Zn Lactate	4.5 ± 1.4 ^b^	24.7 ± 5.6		21.0 ± 9.0	
100 mM	ZnCl_2_	1.3 ± 0.4	9.6 ± 5.0		16.7 ± 7.5	
	Zn Lactate	0.9 ± 0.3	5.1 ± 1.6		12.4 ± 5.6	

**Table 2:** Mean concentration of Co, Cu, Mn, Ni and Zn in the root and shoot tissues of wheat grown with zinc chloride or zinc lactate. Control samples did not receive any zinc supplementation. Different letters indicate a statistically significant difference between treatment concentrations and control (*p* < 0.05; Fisher’s LSD Test). The “†” symbol indicates that the mean was derived from one or more replicates with metal concentrations below the instrument detection limit (DL) and were substituted with values equal to the DL/2. Superscripts “x” and “y” denote a significant difference between zinc chloride and zinc lactate (*p* < 0.05; simple *t*-test). Standard error is denoted in parenthesis; *n* = 5. NS: not significant.

Metal	Root		Shoot	
	**ZnCl_2_**	**Zn Lactate**	**ZnCl_2_**	**Zn Lactate**
**Cobalt (ng g^−1^)**				
Control	35.0 (0.0) a †	35.0 (0.0) a †	30.0 (5.0) a †	30.0 (5.0) a †
0.343 mM	35.0 (0.0) a †	102.8 (67.8) a †	36.9 (1.9) a †	43.2 (6.0) a †
3.43 mM	71.7 (36.7) a †	173.3 (138.3) a †	468.8 (403.4) a †	54.8 (25.9) a †
*p* value	NS	NS	NS	NS
LSD_0.05_	65.3	274.0	717.7	48.2
**Copper (ng g^−1^)**				
Control	2.3 (0.2) a †	2.3 (0.2) a †	6.0 (1.4) a †	6.0 (1.4) a †
0.343 mM	5.0 (1.9) a †	4.9 (1.7) a †	3.2 (0.8) ab †	2.4 (0.7) b †
3.43 mM	2.5 (0.0) a †	3.7 (1.2) a †	2.5 (0.0) b †	2.5 (0.0) b †
*p* value	NS	NS	0.04	0.02
LSD_0.05_	3.4	3.8	2.9	2.8
**Manganese (ng g^−1^)**				
Control	181.2 (25.4) a	181.2 (25.4) a	71.5 (8.3) a	71.5 (8.3) a
0.343 mM	57.6 (5.3) b	44.6 (3.1) b	43.0 (2.0) a	39.4 (2.5) b
3.43 mM	53.0 (9.7) b	60.7 (11.2) b	52.0 (18.1) a	31.7 (2.6) b
*p* value	<0.001	<0.001	NS	<0.001
LSD_0.05_	49.3	49.7	35.6	16.2
**Nickel (ng g^−1^)**				
Control	3.3 (2.0) a †	3.3 (2.0) a †	1.3 (0.6) a †	1.3 (0.6) a †
0.343 mM	3.5 (1.9) a †	10.2 (4.8) a †	0.8 (0.5) a †	1.8 (0.7) a †
3.43 mM	5.7 (3.5) a †	5.6 (2.9) a †	20.9 (17.8) a †	2.6 (1.1) a †
*p* value	NS	NS	NS	NS
LSD_0.05_	7.9	10.6	31.7	2.6
**Zinc (ng g^−1^)**				
Control	670.3 (42.6) c	670.3 (42.6) b	101.2 (12.7) b	101.2 (12.7) b
0.343 mM	5576.6 (438.1) b	8129.7 (1089.1) a	717.3 (122.0) ab	1409.2 (720.4) ab
3.43 mM	11,975.6 (1131.3) a^y^	6729.1 (1842.2) a^x^	5329.2 (2908.2) a	2157.0 (448.9) a
*p* value	<0.001	0.002	NS	0.03
LSD_0.05_	2159.6	3807.9	5178.4	1510.2

**Table 3:** Plant growth parameter results for manganese (Mn) treatments. After 13 days of growth, wheat seedlings were harvested, and shoot length was measured along with fresh weight (FW) and dry weight (DW) of both root and shoot tissue. A *t*-test was used to identify differences between manganese chloride and manganese lactate treatments within each treatment level for each plant growth parameter, where superscripts “a” and “b” denote treatment differences of *p* < 0.05. ±: standard deviation.

Treatment Conc.	Treatment	Shoot Length (cm)	Shoot FW (mg)	Shoot DW (mg)	Root FW (mg)	Root DW (mg)
0 mM	Control	34.0 ± 2.0	297.4 ± 64.6	32.1 ± 7.5	122.4 ± 29.3	8.2 ± 2.8
0.343 mM	MnCl_2_	24.5 ± 12.7	202.0 ± 105.4	22.4 ± 11.4	81.0 ± 34.6	5.5 ± 2.8
	Mn Lactate	28.9 ± 2.0	262.8 ± 47.1	26.9 ± 4.5	114.88 ± 23.3	8.3 ± 2.1
3.43 mM	MnCl_2_	18.3 ± 7.7	147.6 ± 46.1 ^a^	21.5 ±8.1	80.2 ± 18.2	5.5 ± 1.6
	Mn Lactate	22.9 ± 2.9	209.8 ± 21.7 ^b^	29.2 ± 3.8	66.6 ± 14.8	4.1 ± 0.9
34.3 mM	MnCl_2_	12.9 ± 4.1	51.4 ± 27.7		22.0 ± 6.2	
	Mn Lactate	11.2 ± 3.2	41.8 ± 12.3		24.5 ± 8.1	
100 mM	MnCl_2_	2.8 ± 0.5	11.6 ± 5.6		14.8 ± 7.7	
	Mn Lactate	4.9 ± 2.4	19.0 ± 9.9		24.8 ± 9.9	

**Table 4:** Mean concentration of Co, Cu, Mn, Ni and Zn in the root and shoot tissues of wheat grown with manganese chloride or manganese lactate. Control samples did not receive any manganese supplementation. Different letters indicate a statistically significant difference between treatment concentrations and control (*p* < 0.05; Fisher’s LSD Test). The “†” symbol indicates that the mean was derived from one or more replicates with metal concentrations below the instrument detection limit (DL) and were substituted with values equal to the DL/2. No significant differences in tissue metal concentrations were detected between manganese chloride and manganese lactate at any concentration for any metal analyzed (*p* < 0.05; simple *t*-test). Standard error is denoted in parenthesis; *n* = 5. NS: not significant.

Metal	Root		Shoot	
	**MnCl_2_**	**Mn Lactate**	**MnCl_2_**	**Mn Lactate**
**Cobalt (ng g**^−1^)				
Control	356.4 (87.2) a †	356.4 (87.2) a †	104.6 (29.3) a	104.6 (29.3) a
0.343 mM	383.5 (168.0) a †	414.3 (93.5) a	210.2 (131.1) a †	127.3 (28.9) a
3.43 mM	388.0 (51.0) a	493.0 (221.6) a †	187.8 (55.8) a	136.2 (32.1) a †
*p* value	NS	NS	NS	NS
LSD_0.05_	348.8	455.1	258.8	92.9
**Copper (ng g**^−1^)				
Control	2.5 (0.0) a †	2.5 (0.0) a †	2.5 (0.0) a †	2.5 (0.0) a †
0.343 mM	2.5 (0.0) a †	2.5 (0.0) a †	2.6 (0.1) a †	2.8 (0.3) a †
3.43 mM	70.5 (68.0) a †	2.5 (0.0) a †	2.1 (0.4) a †	3.8 (1.3) a †
*p* value	NS	0.01	NS	NS
LSD_0.05_	121.0	<0.001	0.7	2.3
**Manganese (ng g^−1^)**				
Control	59.6 (7.5) c	59.6 (7.5) c	34.8 (3.1) b	34.8 (3.1) b
0.343 mM	2536.3 (399.5) b	2260.8 (412.6) b	1648.8 (181.1) b	1251.8 (74.5) b
3.43 mM	9364.0 (965.5) a	11,235.9 (1025.4) a	8076.7 (3230.5) a	3248.9 (995.3) a †
*p* value	<0.001	<0.001	0.02	0.006
LSD_0.05_	1858.9	1966.3	5756.1	1775.6
**Nickel (ng g^−^**^1^)				
Control	16.5 (3.2) a	16.5 (3.2) a	5.3 (1.1) a	5.3 (1.1) a
0.343 mM	21.6 (4.1) a	14.9 (4.0) a	8.5 (6.6) a †	3.8 (0.3) a
3.43 mM	46.1 (31.7) a	16.9 (5.1) a	4.4 (1.2) a	4.0 (0.6) a
*p* value	NS	NS	NS	NS
LSD_0.05_	57.1	12.8	12.1	2.3
**Zinc (ng g^−1^)**				
Control	407.9 (62.1) a	407.9 (62.1) a	56.4 (7.1) a	56.4 (7.1) ab
0.343 mM	287.4 (125.3) a †	336.6 (84.5) a	165.4 (130.2) a †	64.6 (11.4) a
3.43 mM	456.8 (204.1) a	99.3 (62.3) b †	58.5 (27.1) a	30.8 (8.4) b
*p* value	NS	0.02	NS	NS
LSD_0.05_	440.2	217.0	440.2	28.2

**Table 5:** Plant growth parameter results for copper (Cu) treatments. After 13 days of growth, wheat seedlings were harvested, and shoot length was measured along with fresh weight (FW) and dry weight (DW) of both root and shoot tissue. A *t*-test was used to identify differences between copper chloride and copper lactate within each treatment level for each plant growth parameter, where superscripts “a” and “b” denote treatment differences of *p* < 0.05. ±: standard deviation.

Treatment Conc.	Treatment	Shoot Length (cm)	Shoot FW (mg)	Shoot DW (mg)	Root FW (mg)	Root DW (mg)
0 mM	Control	35.1 ± 1.7	336.8 ± 66.5	32.2 ± 5.7	151.2 ± 46.6	9.2 ± 2.8
0.343 mM	CuCl_2_	22.2 ± 4.0	177.0 ± 36.9	22.6 ± 6.4	50.3 ± 6.7	5.4 ± 0.6
	Cu Lactate	24.3 ± 3.7	166.4 ± 47.5	22.9 ± 7.2	42.8 ± 16.2	4.8 ± 1.8
3.43 mM	CuCl_2_	2.3 ± 0.8	12.0 ± 5.2	3.2 ± 2.5	20.6 ± 6.7	2.0 ± 1.0 ^a^
	Cu Lactate	4.2 ± 3.0	23.2 ± 21.1	4.5 ± 5.0	22.7 ± 6.7	3.3 ± 0.4 ^b^
34.3 mM	CuCl_2_	1.5 ±0.5	9.9 ± 4.2		18.6 ± 2.5	
	Cu Lactate	1.4 ± 0.5	6.4 ± 2.2		16.2 ± 6.5	
100 mM	CuCl_2_	1.2 ± 0.3	7.7 ± 4.8		16.7 ± 5.0	
	Cu Lactate	0.9 ± 0.2	7.0 ± 2.9		8.0 ± 8.4	

**Table 6:** Mean concentration of Co, Cu, Mn, Ni and Zn in the root and shoot tissues of wheat grown with copper chloride or copper lactate. Control samples did not receive any copper supplementation. Different letters indicate a statistically significant difference between treatment concentrations and control (*p* < 0.05; Fisher’s LSD Test). The “†” symbol indicates that the mean was derived from one or more replicates with metal concentrations below the instrument detection limit (DL) and were substituted with values equal to the DL/2. No significant differences in tissue metal concentrations were detected between copper chloride and copper lactate of any concentration for any metal analyzed (*p* < 0.05; simple *t*-test). Standard error is denoted in parenthesis; *n* = 5 (“‡” indicates *n* = 4). NS: not significant; NA: not available.

Metal	Root		Shoot	
	**CuCl_2_**	**Cu Lactate**	**CuCl_2_**	**Cu Lactate**
**Cobalt (ng g^−1^**)				
Control	761.3 (215.9) b †	761.3 (215.9) b †	159.5 (50.6) b †	159.5 (50.6) b †
0.343 mM	783.0 (260.8) ab †	738.6 (353.0) b †	298.1 (99.9) ab †	239.8 (90.4) b †
3.43 mM	4372.1 (1994.7) a	2291.5 (390.4) a	2540.0 (1287.0) a †	1419.2 (370.1) a ‡
*p* value	NS	0.008	NS	0.001
LSD_0.05_	3599.4	1012.1	2298.3	NA
**Copper (ng g^−1^)**				
Control	2.5 (0.0) b †	2.5 (0.0) b †	5.8 (1.8) b †	5.8 (1.8) b †
0.343 mM	1801.2 (146.4) b	1846.5 (373.8) b	56.9 (2.4) b	70.4 (16.0) b
3.43 mM	29,219.0 (9319.8) a	14,115.1 (1048.5) a	15,002.2 (4947.8) a	2309.6 (765.8) a ‡
*p* value	0.004	<0.001	0.004	0.002
LSD_0.05_	16,581.9	1980.3	8802.0	NA
**Manganese (ng g^−1^)**				
Control	181.0 (40.1) a	181.0 (40.1) a	82.0 (8.4) a	82.0 (8.4) a
0.343 mM	57.1 (7.8) b	56.9 (7.3) b	37.4 (2.3) b	40.1 (3.0) b
3.43 mM	95.0 (25.7) b	62.9 (8.5) b	104.7 (19.4) a	58.5 (8.7) b ‡
*p* value	0.02	0.005	0.007	0.005
LSD_0.05_	85.8	74.0	37.8	NA
**Nickel (ng g^−1^)**				
Control	12.9 (5.5) b	12.9 (5.5) ab	1.7 (0.3) a	1.7 (0.3) a
0.343 mM	7.3 (2.1) b †	3.5 (2.1) b †	2.4 (0.6) a †	2.2 (0.8) a †
3.43 mM	40.2 (11.2) a	20.8 (5.0) a	20.2 (12.9) a †	9.1 (4.6) a †‡
*p* value	0.017	0.05	NS	NS
LSD_0.05_	22.5	13.8	22.9	NA
**Zinc (ng g^−1^)**				
Control	1013.2 (124.0) a	1013.2 (124.0) a	148.6 (6.1) a	148.6 (6.1) ab
0.343 mM	245.9 (61.2) b †	230.0 (84.4) b †	81.2 (10.7) a	75.3 (16.8) b
3.43 mM	581.7 (182.1) b	269.0 (14.1) b	190.8 (82.2) a †	183.7 (60.6) a ‡
*p* value	0.005	<0.001	NS	NS
LSD_0.05_	406.8	268.0	147.8	NA

**Table 7:** Plant growth parameter results for nickel (Ni) treatments. After 13 days of growth, wheat seedlings were harvested, and shoot length was measured along with fresh weight (FW) and dry weight (DW) of both root and shoot tissue. A *t*-test was used to identify differences between nickel chloride and nickel lactate treatments within each treatment level for each plant growth parameter, where superscripts “a” and “b” denote treatment differences of *p* < 0.05. ±: standard deviation.

Treatment Conc.	Treatment	Shoot Length (cm)	Shoot FW (mg)	Shoot DW (mg)	Root FW (mg)	Root DW (mg)
0 mM	Control	32.4 ± 3.4	353.7 ± 41.8	35.6 ± 5.8	165.9 ± 36.5	10.6 ± 2.2
0.343 mM	NiCl_2_	19.5 ± 4.5 ^a^	139.4 ± 39.9 ^a^	22.9 ± 7.4	46.5 ± 15.5	5.4 ± 1.4
	Ni Lactate	27.1 ± 4.1 ^b^	221.7 ± 63.4 ^b^	27.8 ± 5.6	73.3 ± 25.7	6.3 ± 1.8
3.43 mM	NiCl_2_	3.5 ± 1.1	29.2 ± 14.7	6.8 ± 2.3	23.2 ± 9.7	3.9 ± 1.5
	Ni Lactate	3.9 ± 1.3	25.4 ± 7.2	6.7 ± 2.9	23.3 ± 6.3	4.1 ± 0.8
34.3 mM	NiCl_2_	2.1 ± 0.6 ^a^	12.8 ± 4.9		24.7 ± 11.2	
	Ni Lactate	3.4 ± 0.8 ^b^	26.9 ± 11.2		36.3 ± 11.9	
100 mM	NiCl_2_	2.3 ± 0.3 ^a^	12.6 ± 2.1 ^a^		22.7 ± 3.5	
	Ni Lactate	3.3 ± 0.4 ^b^	22.9 ± 1.7 ^b^		30.1 ± 12.3	

**Table 8:** Mean concentration of Co, Cu, Mn, Ni and Zn in the root and shoot tissues of wheat grown with nickel chloride or nickel lactate. Control samples did not receive any nickel supplementation. Different letters indicate a statistically significant difference between treatment concentrations and control (*p* < 0.05; Fisher’s LSD Test). The “†” symbol indicates that the mean was derived from one or more replicates with metal concentrations below the instrument detection limit (DL) and were substituted with values equal to the DL/2. Superscripts “x” and “y” denote a significant difference between nickel chloride and nickel lactate at the specified concentration (*p* < 0.05; simple *t*-test). Standard error is denoted in parenthesis; *n* = 5. NS: not significant; NA: not available.

Metal	Root		Shoot	
	**NiCl_2_**	**Ni Lactate**	**NiCl_2_**	**Ni Lactate**
**Cobalt (ng g^−1^)**				
Control	183.2 (29.6) a	183.2 (29.6) a	35.0 (0.0) a †	35.0 (0.0) a †
0.343 mM	35.0 (0.0) b †	35.0 (0.0) b †	35.0 (0.0) a †	35.0 (0.0) a †
3.43 mM	35.0 (0.0) b †	35.0 (0.0) b †	35.0 (0.0) a †	35.0 (0.0) a †
*p* value	<0.001	<0.001	NA	NA
LSD_0.05_	52.6	52.6	NA	NA
**Copper (ng g^−1^)**				
Control	2.5 (0.0) a †	2.5 (0.0) a †	2.5 (0.0) a †	2.5 (0.0) a †
0.343 mM	2.5 (0.0) a †	6.5 (4.0) a †	2.5 (0.0) a †	2.5 (0.0) a †
3.43 mM	2.5 (0.0) a †	2.5 (0.0) a †	2.5 (0.0) a †	2.5 (0.0) a †
*p* value	NA	NS	NA	NA
LSD_0.05_	NA	7.1	NA	NA
**Manganese (ng g^−1^)**				
Control	134.3 (12.1) a	134.3 (12.1) a	57.9 (4.6) a	57.9 (4.6) a
0.343 mM	32.6 (7.9) b	95.8 (22.4) ab	60.6 (29.1) a	66.9 (16.5) a
3.43 mM	10.4 (3.0) b †	38.7 (23.6) b	17.9 (3.5) a	21.9 (2.7) b
*p* value	<0.001	0.02	NS	0.02
LSD_0.05_	26.3	61.8	52.8	30.8
**Nickel (ng g^−1^)**				
Control	0.25 (0.0) b †	0.25 (0.0) b †	0.25 (0.0) b †	0.25 (0.0) b †
0.343 mM	1086.9 (64.7) b	1286.1 (192.3) a	598.6 (32.9) b^y^	252.0 (28.4) b^x^
3.43 mM	4956.9 (662.7) a^y^	993.7 (132.4) a^x^	4340.3 (961.6) a	2287.1 (531.1) a
*p* value	<0.001	<0.001	<0.001	<0.001
LSD_0.05_	1184.5	415.3	1711.8	946.2
**Zinc (ng g^−1^)**				
Control	385.1 (29.5) a	385.1 (29.5) a	90.8 (7.7) a	90.8 (7.7) a
0.343 mM	330.8 (29.5) a	360.1 (42.5) a	62.7 (4.9) a	61.0 (3.9) a
3.43 mM	415.6 (90.7) a	291.1 (12.9) a	120.8 (48.8) a	89.3 (15.4) a
*p* value	NS	NS	NS	NS
LSD_0.05_	177.7	94.9	88.4	31.4

**Table 9:** Plant growth parameter results for cobalt (Co) treatments. After 13 days of growth, wheat seedlings were harvested, and shoot length was measured along with fresh weight (FW) and dry weight (DW) of both root and shoot tissue. A *t*-test was used to identify differences between cobalt chloride and cobalt lactate treatments within each treatment level for each plant growth parameter, where superscripts “a” and “b” denote treatment differences of *p* < 0.05. ±: standard deviation.

Treatment Conc.	Treatment	Shoot Length (cm)	Shoot FW (mg)	Shoot DW (mg)	Root FW (mg)	Root DW (mg)
0 mM	Control	28.6 ± 8.6	272.2 ± 64.5	28.1 ± 5.3	91.3 ± 2.9	8.1 ± 2.9
0.343 mM	CoCl_2_	16.5 ± 4.0	132.4 ± 47.1	16.7 ± 5.0	58.7 ± 12.3	6.3 ± 1.3
	Co Lactate	20.1 ± 2.7	177.9 ± 40.1	20.6 ± 4.3	70.8 ± 17.9	6.5 ± 1.8
3.43 mM	CoCl_2_	8.1 ± 1.5	46.6 ± 10.9	7.8 ± 2.9	28.5 ± 5.9	4.3 ± 1.1 ^a^
	Co Lactate	7.0 ± 1.5	46.5 ± 7.6	8.8 ± 2.3	29.3 ± 4.6	6.4 ± 1.4 ^b^
34.3 mM	CoCl_2_	2.1 ± 0.3 ^a^	10.7 ± 2.5		21.3 ± 2.2	
	Co Lactate	2.6 ± 0.4 ^b^	11.4 ± 3.7		19.6 ± 7.9	
100 mM	CoCl_2_	1.0 ± 0.3 ^a^	7.9 ± 1.5		18.3 ± 5.7	
	Co Lactate	1.4 ± 0.2 ^b^	8.0 ± 2.2		19.6 ± 4.4	

**Table 10:** Mean concentration of Co, Cu, Mn, Ni and Zn in the root and shoot tissues of wheat grown with cobalt chloride or cobalt lactate. Control samples did not receive any cobalt supplementation. Different letters indicate a statistically significant difference between treatment concentrations and control (*p* < 0.05; Fisher’s LSD Test). The “†” symbol indicates that the mean was derived from one or more replicates with metal concentrations below the instrument detection limit (DL) and were substituted with values equal to the DL/2. No significant differences in tissue metal concentrations were detected between cobalt chloride and cobalt lactate at any concentration for any metal analyzed (*p* < 0.05; simple *t*-test). Standard error is denoted in parenthesis; *n* = 5. NS: not significant.

Metal	Root		Shoot	
	**CoCl_2_**	**Co Lactate**	**CoCl_2_**	**Co Lactate**
**Cobalt (ng g^−1^)**				
Control	380.5 (181.9) b †	380.5 (181.9) c †	50.2 (15.2) b †	50.2 (15.2) b †
0.343 mM	2578.7 (204.5) b	1984.7 (188.2) b	836.8 (95.5) b	799.0 (103.6) b
3.43 mM	9365.0 (1340.5) a	7800.2 (678.3) a	3560.0 (1053.4) a	4398.3 (1063.5) a
*p* value	<0.001	<0.001	0.004	<0.001
LSD_0.05_	2433.9	1293.4	1881.8	1901.1
**Copper (ng g^−1^)**				
Control	28.0 (10.0) a	28.0 (10.0) a	5.5 (2.1) a †	5.5 (2.1) a †
0.343 mM	16.4 (8.6) ab †	3.3 (0.8) b †	2.5 (0.0) a †	2.1 (0.4) a †
3.43 mM	2.5 (0.0) b †	2.5 (0.0) b †	2.5 (0.0) a †	2.5 (0.0) a †
*p* value	NS	0.01	NS	NS
LSD_0.05_	23.4	17.8	3.8	3.9
**Manganese (ng g^−1^)**				
Control	145.7 (26.5) a	145.7 (26.5) a	69.0 (4.9) a	69.0 (4.9) a
0.343 mM	54.3 (9.7) a	46.2 (6.5) b	48.6 (3.9) b	51.0 (2.6) b
3.43 mM	40.4 (8.8) b †	48.6 (6.7) b	38.8 (2.9) b	50.9 (4.7) b
*p* value	0.002	0.001	<0.001	0.01
LSD_0.05_	52.5	49.9	12.2	12.9
**Nickel (ng g^−1^)**				
Control	119.4 (41.1) a	119.4 (41.1) a	6.0 (1.2) a	6.0 (1.2) a
0.343 mM	43.3 (43.0) ab †	0.25 (0.0) b †	6.2 (5.9) a †	0.25 (0.0) b †
3.43 mM	0.25 (0.0) b †	0.25 (0.0) b †	0.25 (0.0) a †	0.25 (0.0) b †
*p* value	NS	0.005	NS	<0.001
LSD_0.05_	105.8	73.1	10.7	2.1
**Zinc (ng g^−1^)**				
Control	249.6 (91.8) a †	249.6 (91.8) a †	69.7 (14.6) a	69.7 (14.6) a
0.343 mM	98.8 (14.5) ab	117.0 (16.1) ab	45.5 (8.8) ab	61.5 (7.5) ab
3.43 mM	60.7 (26.9) b	58.5 (16.5) b †	20.5 (7.8) b †	30.9 (9.5) b †
*p* value	NS	NS	0.02	NS
LSD_0.05_	172.2	168.4	33.3	33.7

## Supplementary Materials

The following are available online at https://www.mdpi.com/article/10.3390/biom11081085/s1, Table S1: Volume of modified Hoagland’s nutrient solution (NS), water, and 0.229 M metal lactate or metal chloride solution added to each culture vessel for each selected treatment, Table S2: Final nutrient concentrations in modified Hoagland’s nutrient solution, Figure S1: Set up and growth of the wheat cultivation vessel experiment to measure uptake and toxicity of metal chlorides and metal lactates, Figure S2: Representative dose response of 16-day old wheat (*Triticum aestivum*) plants to zinc chloride (ZnCl_2_) and zinc lactate.
