# Supplementary material for: Plant Uptake of Lactate-Bound Metals: A Sustainable Alternative to Metal Chlorides
Source: Biomolecules. 2021 Jul 23;11(8):1085. doi: 10.3390/biom11081085 (PMC8391765; doi:10.3390/biom11081085)
Supplement: Supplementary file 1 [file biomolecules-11-01085-s001.zip › biomolecules-1259242-supplementary.pdf]

Supplemental Tables and Figures

Plant Uptake of Lactate-Bound Metals: A Sustainable Alternative to Metal Chlorides

Lee J. Opdahl [1], Ricky W. Lewis [2], Lee A. Kalcsits[3], Tarah S. Sullivan[1,*] and Karen A. Sanguinet [1,*]

[1] Department of Crop and Soil Sciences, Washington State University, Pullman, WA, USA; lee.opdahl@wsu.edu (L.J.O.)

[2] Department of Tropical Plant and Soil Sciences, University of Hawai'i at Mānoa, Honolulu, HI, USA; ricky.w.lewis@gmail.com

[3] Tree Fruit Research and Extension Center, Washington State University, Wenatchee, WA, USA; lee.kalcsits@wsu.edu

* Correspondence: t.sullivan@wsu.edu (T.S.S.), Tel.: 509-335-4837; karen.sanguinet@wsu.edu (K.A.S.), Tel.: 509-335-3662

Table S1. Volume of modified Hoagland's nutrient solution (NS), water, and 0.229 M metal lactate or metal chloride solution added to each cultivation vessel for each selected treatment.

Treatment	Hoagland's NS (mL)	Water (mL)	Metal Solution (mL)	*n*
0.343 mM	12.5	37.43	0.0749	ML = 5<br>MC = 5
3.43 mM	12.5	36.45	0.749	ML = 5<br>MC = 5
34.3 mM	12.5	30.01	7.49	ML = 5<br>MC = 5
100 mM	12.5	15.76	21.83	ML = 5<br>MC = 5
Control (0 mM)	12.5	37.5	0	ML = 5<br>MC = 5

The number of replicates (*n*) for each metal lactate (ML) and metal chloride (MC) treatment and the control are indicated in the far-right column.

Table S2. Final nutrient concentrations in modified Hoagland's nutrient solution.

Nutrient	Conc. (mM)
$NH_4H_2PO_4$	0.25
KNO_3	1.5
$Ca(NO_3)_2 \cdot 4H_2O$	1.0
$MgSO_4 \cdot 7H_2O$	0.5
H_3BO_3	$1.16 x 10^{-2}$
$MnCl_2 \cdot 4H_2O$	$2.29 x 10^{-3}$
$ZnSO_4 \cdot 7H_2O$	$1.9 x 10^{-4}$
$CuSO_4$	$1.3 x 10^{-4}$
Fe-EDTA	$1.12 x 10^{-2}$
$(NH_4)_6Mo_7O_{24} \cdot 4H_2O)$	$8.09 x 10^{-6}$
MES buffer	10

Concentrations in mM represent the final concentrations of each nutrient in the cultivation vessel solution. The nutrients that were added as metal lactate and metal chloride treatments that are also included in Hoagland's nutrient solution (i.e., Zn, Cu, and Mn) were excluded from the Hoagland's solution.

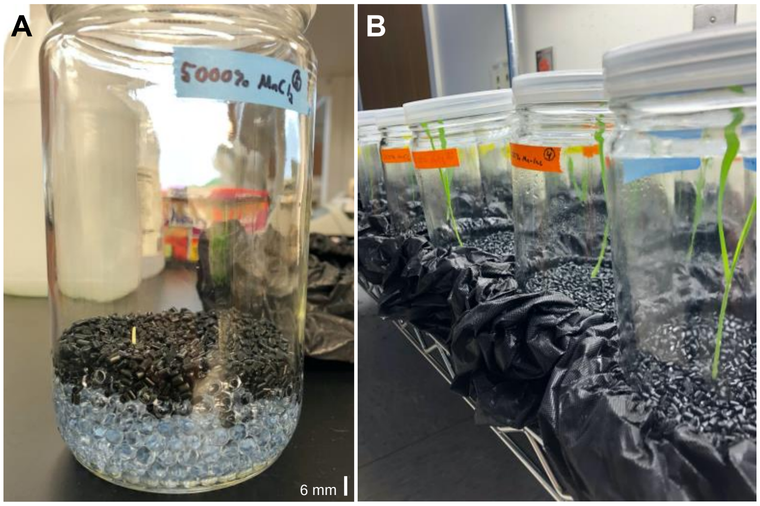

Figure S1. Set up and growth of the wheat cultivation vessel experiment to measure uptake and toxicity of metal chlorides and metal lactates. (A) A sample cultivation vessel after sowing a 3-day old wheat seedling within the 6 mm glass bead physical support matrix and covered with black polypropylene pellets to prevent excessive light exposure on seedling roots. (B) Growth at 13 days after sowing with black plastic covers on the bottom exterior portion of each vessel to further inhibit light exposure on roots. Scale bar equals 6 mm.

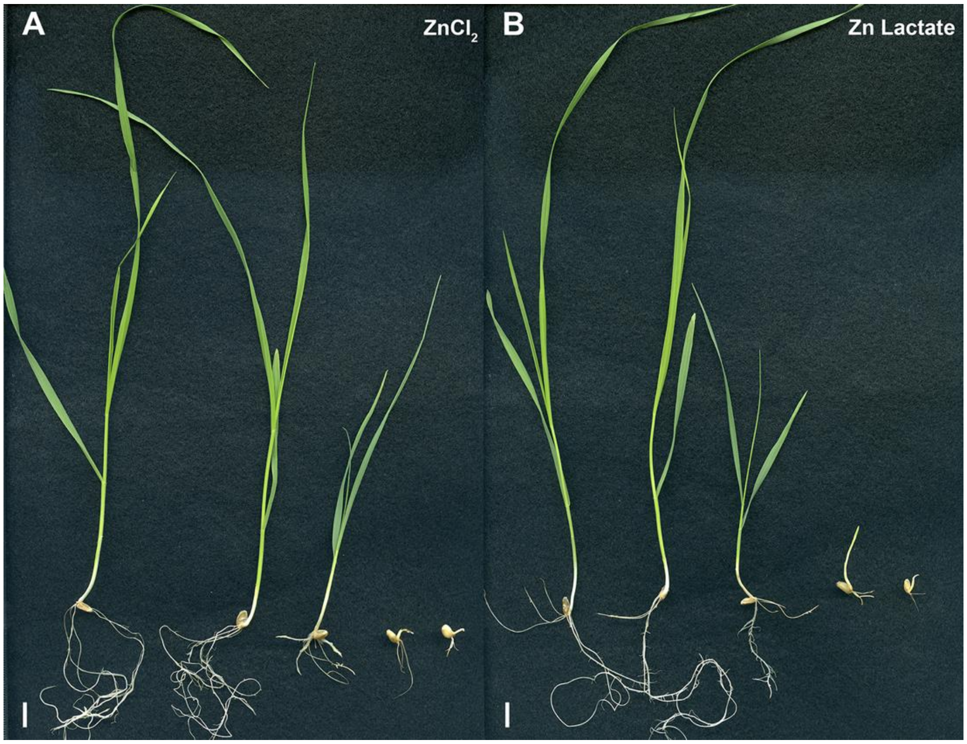

Figure S2. Representative dose response of 16-day old wheat (*Triticum aestivum*) plants to zinc chloride (A) and zinc lactate (B). Individual plants were treated with the following concentrations (left to right in each picture): 0, 0.343, 3.43, 34.3, and 100 mM zinc chloride ($ZnCl_2$) or zinc lactate, respectively. Scale bar equals 1 cm. Images were obtained on a Epson V700 flatbed scanner at 600 dpi.
